# Extracellular volume fraction and native T1 mapping in diabetic cardiomyopathy: a comprehensive meta-analysis

**DOI:** 10.1186/s12872-025-04496-z

**Published:** 2025-02-01

**Authors:** Ahmed Marey, Ali Alabdullah, Hossam Ghorab, Fatima Ali, Jawdat Abdulla, Akhil Narang, Muhammad Umair

**Affiliations:** 1 Alexandria University Faculty of Medicine, Champollion street،, Al Mesallah Sharq, Al Attarin, Alexandria Governorate, Alexandria, 5372066 Egypt; 2 Department of Surgery, Cleveland Clinic Abu Dhabi, Abu Dhabi, United Arab Emirates; 3 CMH Lahore Medical College, Lahore, Pakistan; 4 Department of Cardiology, Amager and Hvidovre Hospital, Hvidovre, Denmark; 5 Department of Cardiology, Northwestern University, Evanston, IL USA; 6 Russell H. Morgan Department of Radiology and Radiological Sciences, The Johns Hopkins Hospital, Baltimore, MD USA

**Keywords:** Diabetes, Myocardial fibrosis, Extracellular volume fraction, T1 mapping, Cardiovascular magnetic resonance, Diabetic cardiomyopathy

## Abstract

**Background:**

Type 2 diabetes mellitus (T2DM) is associated with myocardial fibrosis (MF), a major contributor to adverse cardiovascular outcomes. Cardiovascular magnetic resonance (CMR), specifically extracellular volume fraction (ECV) and native T1 mapping, offers a non-invasive approach to quantify MF. This study aims to evaluate the utility of ECV and native T1 mapping as biomarkers for cardiac fibrosis and to assess their relationship with diabetes severity, measured by hemoglobin A1C (HbA1C), in patients with T2DM.

**Methods:**

A systematic review and meta-analysis were conducted following PRISMA guidelines. Comprehensive searches identified 19 eligible studies comprising 4,117 participants. Weighted mean differences (WMDs) were calculated for ECV and native T1 values between diabetic and non-diabetic groups. Meta-regression assessed the correlation between ECV and HbA1C. Sensitivity and subgroup analyses were performed to explore heterogeneity.

**Results:**

Diabetic patients exhibited significantly higher ECV values than controls (WMD: 2.17; 95% CI: 1.32–3.02), consistent across subgroups excluding cardiac comorbidities (WMD: 2.02; 95% CI: 0.74–3.31). HbA1C levels were also significantly elevated in diabetics (WMD: 1.78; 95% CI: 1.37–2.19). However, no significant difference in native T1 values was observed (WMD: 13.40; 95% CI: -13.98–40.79). Meta-regression revealed no significant correlation between ECV and HbA1C, potentially due to limited data and high heterogeneity (I²: 93.37%).

**Conclusions:**

ECV is a promising marker for quantifying MF in T2DM, demonstrating significant differences between diabetics and controls. The lack of correlation between ECV and HbA1C underscores the complexity of MF in diabetes and highlights the need for further research. Future studies with standardized protocols are essential to validate these findings and refine the use of CMR in diabetic cardiomyopathy.

**Supplementary Information:**

The online version contains supplementary material available at 10.1186/s12872-025-04496-z.

**Table:** 

Research Insights	Summary
What is currently known about this topic?	- Diabetic cardiomyopathy involves myocardial fibrosis as a key pathophysiological feature.
	- ECV and native T1 mapping by CMR are non-invasive methods to quantify myocardial fibrosis.
	- Myocardial fibrosis is linked to adverse cardiovascular outcomes in diabetes patients.
What is the key research question?	How do CMR-derived ECV and native T1 mapping relate to glycemic control in diabetic patients?
What is new?	- ECV is significantly elevated in diabetics vs. non-diabetics, indicating diffuse fibrosis.
	- Native T1 mapping showed no significant difference between diabetics and controls.
	- No correlation was found between ECV and HbA1C, underscoring complex fibrosis mechanisms.
How might this study influence clinical practice?	ECV can guide early detection and management of myocardial fibrosis in diabetic populations.

## Introduction

Type 2 diabetes is a common global chronic disease that can lead to myocardial dysfunction independent of coronary artery disease [1, 2]. While the exact underlying pathogenesis is likely multifactorial [1–3], it ultimately leads to accelerated cellular apoptosis and necrosis, causing increased perivascular and diffuse interstitial fibrosis in the myocardium [2]. Notably, prior studies have provided histological evidence of heightened diffuse microscopic fibrosis in the myocardium of diabetic patients [4, 5].

Myocardial fibrosis (MF) is closely linked to cardiovascular events, such as heart failure (HF), coronary artery disease (CAD), atrial fibrillation (AF), and peripheral arterial disease (PAD) [6]. The severity of myocardial fibrosis (MF), as measured histologically, has been independently linked to an increased risk of mortality [7, 8]. MF arises from an excessive and disproportionate buildup of collagen in the myocardial extracellular matrix [9, 10], which can lead to reduced myocardial compliance and both diastolic and systolic dysfunction. Additionally, MF can impair impulse propagation, contributing to arrhythmias and conduction abnormalities [11]. Characterized by the expansion of the myocardial interstitium, MF is now regarded as a “preeminent therapeutic target of the 21st century” [12].

Endomyocardial biopsy is the gold standard for fibrosis assessment, but cardiovascular magnetic resonance (CMR) offers a non-invasive alternative. CMR, specifically the extracellular volume fraction (ECV) from T1-mapping, can detect interstitial fibrosis [13]. ECV is a sensitive marker associated with adverse cardiovascular events.

The primary objective of this study is to evaluate the utility of ECV and native T1 mapping as biomarkers for detecting myocardial fibrosis in patients with T2DM. Additionally, we investigate the relationship between these biomarkers and glycemic control, measured by hemoglobin A1C (HbA1C), to better understand the interplay between metabolic dysregulation and cardiac remodeling. This comprehensive meta-analysis synthesizes existing evidence to provide a clearer understanding of the role of CMR-derived parameters in diabetic cardiomyopathy and their potential clinical applications.

## Methods

### Data sources and search strategy

We adhered to a standardized guide for designing, conducting, and publishing systematic reviews and meta-analyses. Reporting was aligned with PRISMA (Preferred Reporting Items for Systematic Reviews and Meta-Analyses) guidelines. We performed a comprehensive search across multiple databases, including EMBASE, Medline (Ovid), Cochrane CENTRAL, Web of Science, and Google Scholar, with the latest update occurring on May 24, 2024. Studies involving animals, conference abstracts, letters to the editor, comments, and editorials were excluded, with no restrictions on language or publication date. We also excluded studies on type 1 diabetes.

### Study selection

Eligible studies met the following inclusion criteria: (1) observational or interventional design; (2) investigation of the association between diabetes status or glycemic control and myocardial fibrosis (MF), measured via CMR T1 mapping; and (3) reporting of effect estimates with 95% confidence intervals, mean with standard deviations, median with interquartile ranges or p-values. Two independent reviewers screened all abstracts, and full-text articles were retrieved for studies that passed screening. Full-text assessments were also conducted by two independent reviewers, and any disagreements were resolved by consulting a third reviewer. A cross-reference search was performed using the bibliographies of included studies, applying the same inclusion criteria.

### Data extraction and quality assessment

Study characteristics such as study design and sample size, along with participant details like age, sex, body mass index (BMI), diabetes status, glycosylated hemoglobin (HbA1C), as well as estimates of myocardial fibrosis (ECV, native T1), were documented using a predesigned data collection form.

The primary outcomes include difference in ECV and native T1 values between diabetics and normal population. Secondary endpoints involve evaluating the correlation between these parameters and glycemic control, measured by hemoglobin A1C (HbA1C).

Two independent reviewers evaluated each study. The quality of the studies was assessed using the Newcastle-Ottawa Scale.

### Data synthesis and analysis

Continuous outcomes were summarized as mean ± standard deviation (SD) to facilitate comparison. We applied Cochrane recommendations in converting median and interquartile range (IQR) to mean and standard deviation (SD). A random effects model was used to pool effect estimates from studies that reported MF using the same modality and measurement scale. Weighed mean difference was used as the effect size to measure the difference between ECV and T1 in diabetics and normal population. Correlation between glycemic control (HbA1C) and CMR parametric mapping (ECV, native T1) were addressed using meta-regression of the pooled effect sizes. Heterogeneity was assessed using the I² statistic: I² ≤25% indicated low heterogeneity, 25–75% moderate, and > 75% high heterogeneity. All statistical analyses were performed using R.

### Subgroup and meta-regression analyses

Subgroups were defined based on key variables, including imaging protocols (e.g., field strength [1.5T vs. 3T] and mapping techniques [MOLLI vs. shMOLLI]). These subgroup definitions were predetermined to account for potential sources of heterogeneity.

For meta-regression, the included variables were selected based on their relevance to the study objectives and data availability. The primary independent variable was HbA1C, representing glycemic control. Other covariates included imaging parameters (e.g., field strength, mapping technique). The meta-regression model aimed to explore the relationship between ECV values (outcome variable) and HbA1C levels, adjusting for potential confounders. Weighted regression coefficients were calculated, and residual heterogeneity (I²) was assessed to determine the model’s explanatory power. Sensitivity analyses were conducted by excluding outlier studies and re-evaluating the regression results.

## Results

### Literature search

**Fig. 1:**
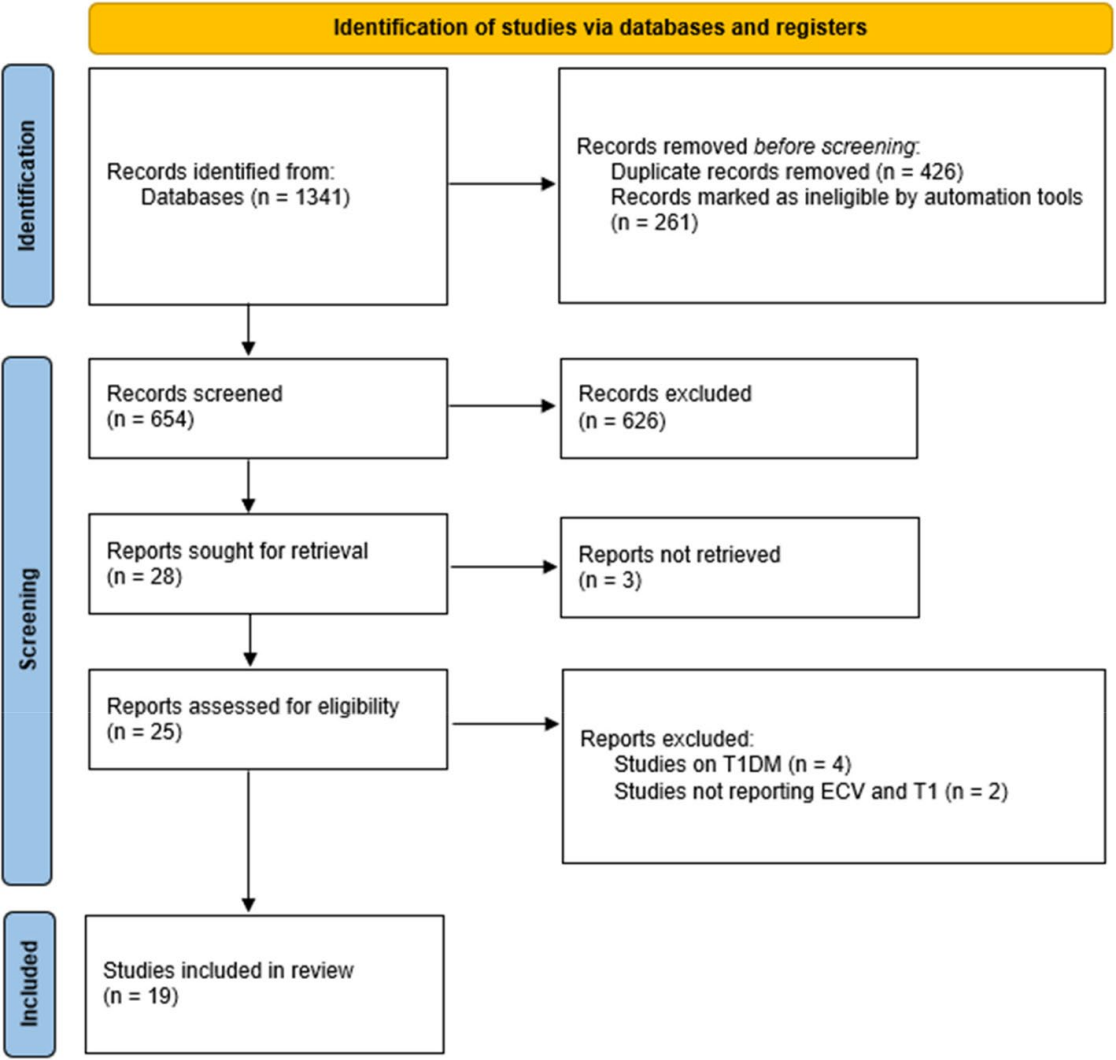
PRISMA Flow Chart

The outcomes of the search strategy are depicted in Fig. 1. Out of 915 unique citations, 19 papers met the inclusion criteria. Key study characteristics are detailed in Table 1. Sample sizes across studies ranged from 33 to 1176 participants, with a combined total population of 4117. The median age of participants was 57.4 years (IQR: 54.35–62.5 years). The studies examined the association between diabetes status, glycemic control (HbA1C), and parametric CMR measures (ECV and T1). The researches were conducted across Europe (*n* = 5), Asia (*n* = 5), North America (*n* = 8), and Australia (*n* = 1). MF was assessed using cardiovascular magnetic resonance (CMR) based native T1 mapping and its derivative extracellular volume percentage (ECV%) These parametric mapping based CMR parameters in this study population were investigated at 1.5-T (*n* = 10) and 3.0-T (*n* = 9) MRI systems. CMR protocol of included studies are summarized in Table 2.

**Table 1:** Summary of included studies

Study (ref)	Country	Study design	*N*	Study Population	Mean Age	Female %
Al-Badri 2018 [14]	USA	Case control	47	Patients from veteran’s hospital	65.8	11
Cao 2018 [15]	China	Case control	82	Patients in tertiary hospital and community controls	54.6	45
Chirinos 2019 [16]	USA	Secondary analysis of baseline data from a previous phase 2 randomized controlled trial (RCT)	53	Subjects with symptomatic HFpEF (LVEF > 50%) were included based on specific clinical criteria (e.g., previous HF hospitalization, elevated NT-proBNP)	64 (median)	24.52
Dennis 2022 [17]	Australia	Case control	67	Patients were recruited from the Royal Prince Alfred Hospital (RPAH) Diabetes Centre – a university teaching hospital, outpatient diabetes service.	61 (median)	39
Gao 2019 [18]	China	Case control	100	Patients in tertiary hospital and healthy volunteers	57.4	59
Gulsin 2019 [19]	United Kingdom	Prospective cohort	96	HFpEF cases from outpatient and inpatient clinics	72.3	51
Jiang 2020 [20]	USA	Prospective cohort	190	Patients from a tertiary hospital and healthy control subjects	55.6	37
Khan 2020 [21]	USA	Case control	442	Patients referred for CMR at tertiary hospital	60.2	52
Kim 2022 [22]	Republic of Korea	Case control	233	Asymptomatic subjects who underwent CMR for health screening at tertiary hospital	54.1	9.8
Kropidlowski 2020 [23]	Germany	Retrospective cohort	63	Patients with well-treated hypertension and healthy volunteers	58.7	49.2
Kucukseymen 2020 [24]	USA	Retrospective cohort	207	Patients with diabetes, obesity, or both and healthy control subjects	55.6	61
Lam 2019 [25]	USA	Case control	37	Patients from endocrinology clinic and community control subjects	49.5	70
Laohabut 2021 [26]	Thailand	Retrospective cohort	739	Patients with known or suspected coronary artery disease who underwent CMR at a tertiary hospital	69.5	50.7
Levelt 2016 [27]	United Kingdom	Prospective observational study	66	Patients with DM in general practice clinics and community control subjects	54.7	51
Liu 2022 [28]	China	Case control	122	Patients who were diagnosed with T2DM in the Department of Endocrinology at tertiary hospital	53.18	41
Shah 2013 [29]	USA	Prospective cohort	33	Obese adolescents in a tertiary hospital and healthy volunteers	16.8	51.5
Storz 2018 [30]	Germany	Case control	343	General population	59.1	31
Swoboda 2017 [31]	United Kingdom	Case control	130	Patients in general practice clinics and community control subjects	60.3	21
Wong 2014 [32]	USA	Case control	1176	Patients referred for CMR at tertiary hospital	54.6	41

**Table 2:** CMR Protocol of included studies

Study (ref)	MRI Field Strength	Vendor	T1 mapping technique
Al-Badri 2018 [14]	1.5	Siemens Medical Solutions, Erlangen, Germany	MOLLI
Cao 2018 [15]	1.5	Siemens Medical Solutions, Erlangen, Germany	MOLLI
Chirinos 2019 [16]	1.5	Siemens Medical Solutions, Erlangen, Germany	MOLLI
Dennis 2022 [17]	1.5	Achieva, Philips Healthcare, Best, The Netherlands	MOLLI
Gao 2019 [18]	3	Skrya; Siemens Medical Solutions, Erlangen, Germany	MOLLI
Gulsin 2019 [19]	3	Siemens Medical Solutions, Erlangen, Germany	shMOLLI
Jiang 2020 [20]	3	Siemens Medical Solutions, Erlangen, Germany	MOLLI
Khan 2020 [21]	Both	Siemens Medical Solutions, Erlangen, Germany	MOLLI
Kim 2022 [22]	1.5	Siemens Healthineers	MOLLI
Kropidlowski 2020 [23]	1.5	Siemens Medical Solutions, Erlangen, Germany	MOLLI
Kucukseymen 2020 [24]	1.5	Siemens Medical Solutions, Erlangen, Germany	MOLLI
Lam 2019 [25]	1.5	Siemens Medical Imaging Solutions, Erlanger, Germany	MOLLI
Laohabut 2021 [26]	3	Siemens Medical Solutions, Erlangen, Germany	shMOLLI
Levelt 2016 [27]	3	Siemens, Germany	shMOLLI
Liu 2022 [28]	3	Skyra, Siemens Medical Solutions	MOLLI
Shah 2013 [29]	3	Siemens Verio, Siemens, Erlangen, Germany	MOLLI
Storz 2018 [30]	3	Siemens AG, Healthcare Sector, Erlangen, Germany	MOLLI
Swoboda 2017 [31]	3	Achieva system, Tesla Philips	MOLLI
Wong 2014 [32]	1.5	Siemens Medical Solutions, Erlangen, Germany	MOLLI

### Quality and risk of bias assesment

The risk of bias in observational studies varied from low (24%), to moderate (34%), to high (42%) (Supplemental Table 1). The quality of the included studies varied significantly, as reflected by their Newcastle-Ottawa Scale (NOS) scores. High-quality studies (NOS scores of 7 or 8, e.g., Jiang 2020, Kucukseymen 2020, Storz 2018) demonstrated robust methodologies and comprehensive reporting, contributing to the reliability of pooled estimates. Conversely, studies rated as poor (e.g., Al-Badri 2018, Lam 2019) often exhibited limitations in selection criteria or comparability, which may have introduced bias.

### Diabetes and ECV & T1

**Fig. 2:**
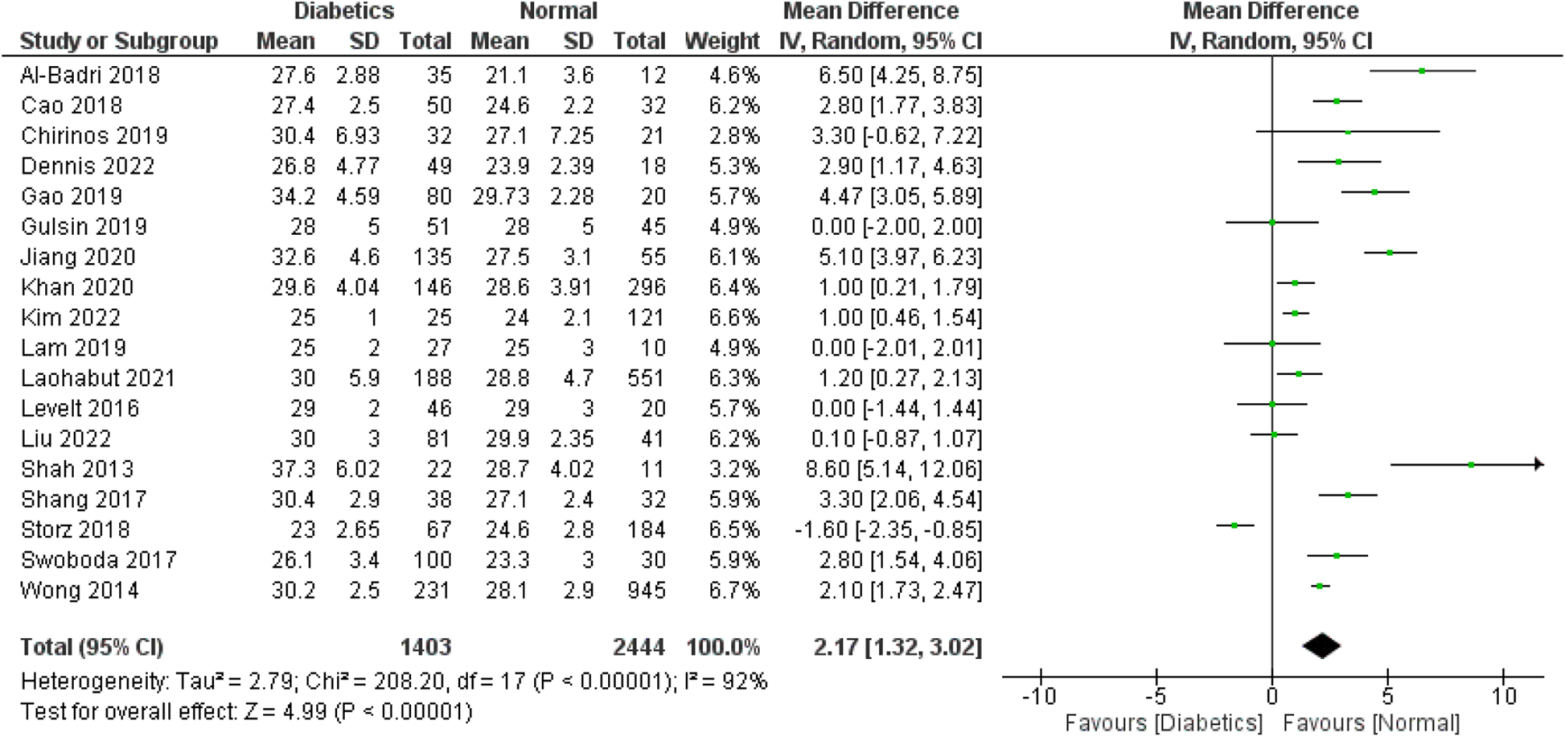
WMD of ECV values between the study arms: Statistically significant difference in ECV was found between diabetics and normal populations (WMD: 2.17; 95% CI: 1.32 to 3.02)

**Fig. 3:**
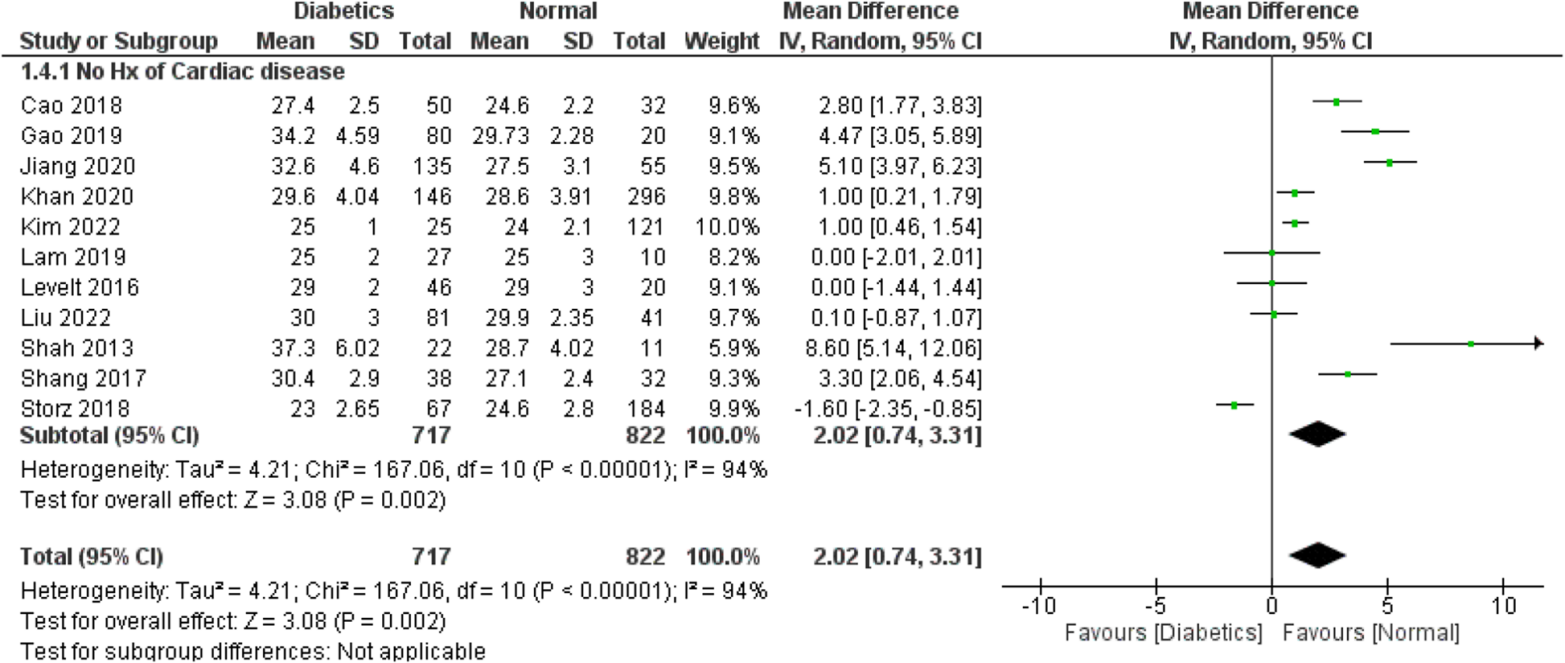
WMD of ECV values between the study arms (Studies with any history of cardiac disease excluded): Statistically significant difference in ECV was found between diabetics and normal populations (WMD: 2.02; 95% CI: 0.74 to 3.31)

**Fig. 4:**
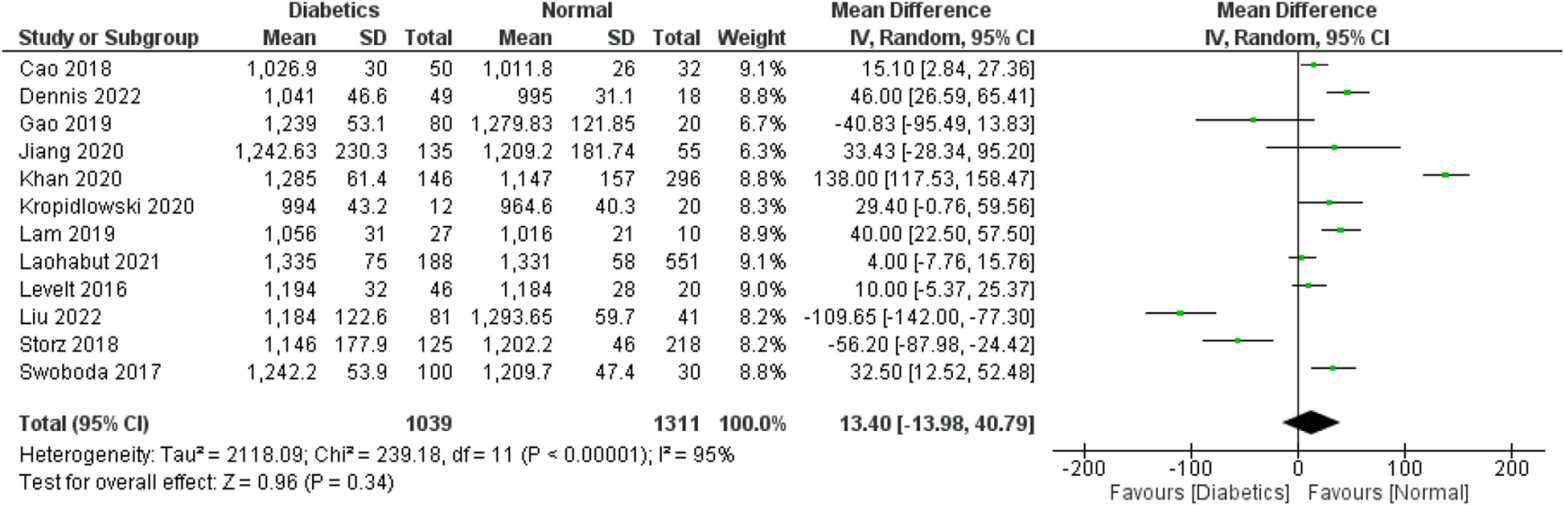
WMD of native T1 values between the study arms: No statistically significant difference in native T1 was found between diabetics and normal populations (WMD: 13.40; 95% CI: −13.98 to 40.79)

**Fig. 5:**
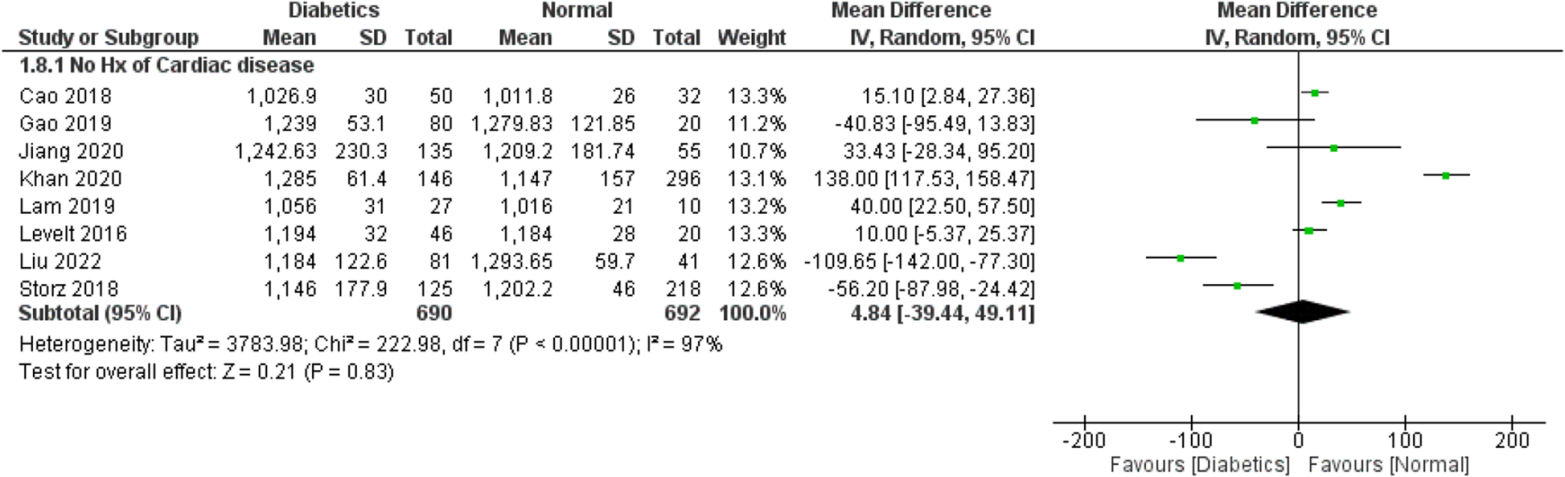
WMD of native T1 values between the study arms (Studies with any history of cardiac disease excluded): No statistically significant difference in native T1 was found between diabetics and normal populations (WMD: 11.88; 95% CI: −17.35 to 41.11)

**Fig. 6:**
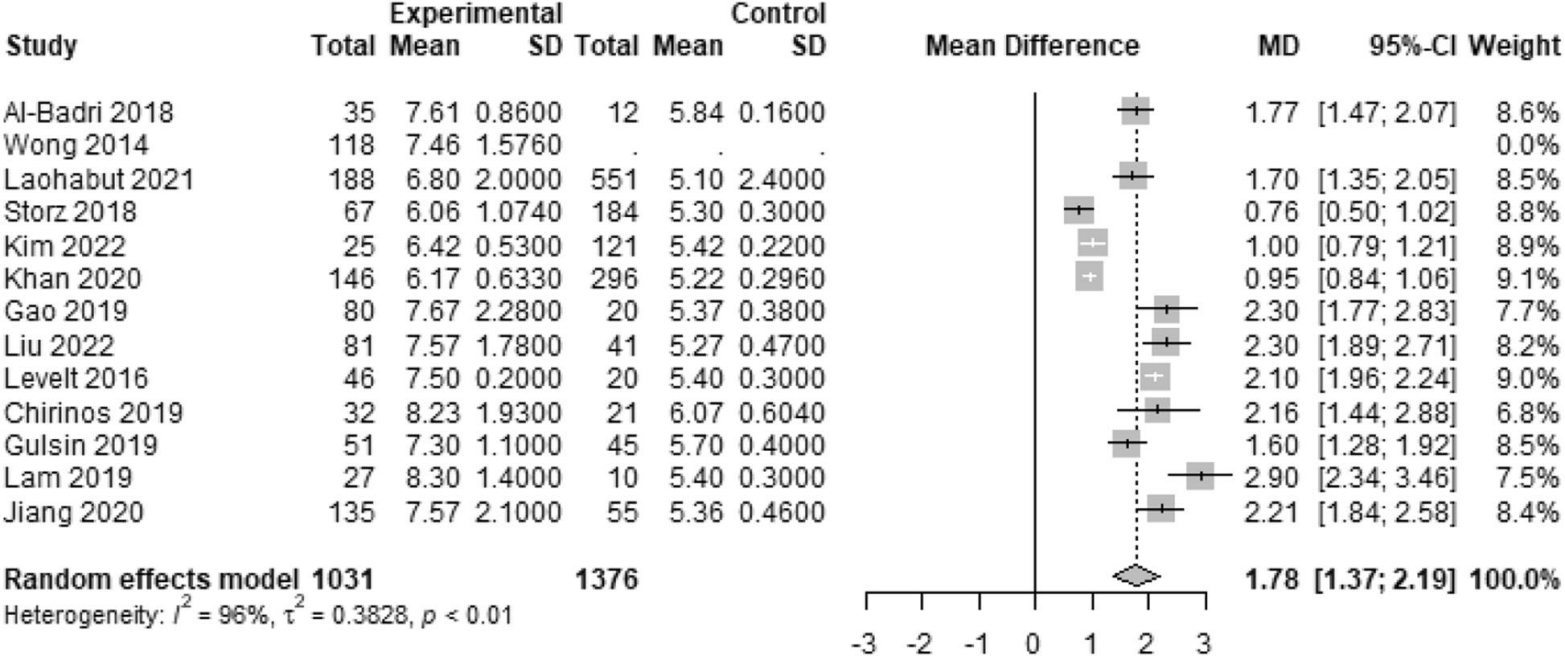
WMD of HbA1C values between the study arms. Statistically significant difference in HbA1C was found between diabetics and normal populations (WMD: 1.78; 95% CI: 1.37 to 2.19)

Figures 2, 3, 4, 5 and 6 investigate if the weighed mean difference (WMD) of the investigated parameters of our study (ECV, native T1, and HbA1C) between the study groups (diabetics VS control subjects).

Figure 2 demonstrates a significantly higher ECV values among the cases compared to the controls (WMD: 2.17; 95% CI: 1.32 to 3.02). We performed a separate analysis for the studies which excluded patients with a history of any cardiac disease or MI which showed the same findings (WMD: 2.02; 95% CI: 0.74 to 3.31) (Fig. 3). The same applies for HbA1C values (Fig. 6), which are expectedly higher among the cases (WMD: 1.78; 95% CI: 1.37 to 2.19). This suggests a potential correlation between ECV and DM control (as estimated by the HbA1C lab values). As for native T1 values (Fig. 4), no statistically significant difference between the two groups was detected (WMD: 13.40; 95% CI: −13.98 to 40.79). Additional analysis of studies excluding patients with a history of any cardiac disease or MI showed the same findings (WMD: 11.88; 95% CI: −17.35 to 41.11) (Fig. 5). However, significant heterogeneity was present in all the analyses.

Table 3 shows results from the meta-regression model fit to assess the correlation between ECV values (outcome variable) and HbA1C (independent predictor variable) while accounting for not only the weighed values for the two parameters, but also their corresponding variance. As noted below, it appears that no significant correlation between ECV and HbA1C could be detected (p-value: 0.5064). The interpretation of these findings should be done with highest caution. One reason for this is the fact that the meta-regression model could potentially be underpowered since only few of the included studies have reported the HbA1C values for the two study arms (Fig. 4). Another reason is the notably high residual heterogeneity (I^2: 93.37%), which suggests that the model selection requires more thoughtful reconsideration.

**Table 3:** A meta regression model assessing the correlation between weighed mean ECV values and HbA1C values

Parameter	Estimate	Standard Error	z-value	*p*-value	Confidence Interval (Lower Bound)	Confidence Interval (Upper Bound)	Significance
**Intercept**	−0.2240	1.9890	−0.1126	0.9103	−4.1224	3.6743	
**HbA1C yi**	0.8823	1.3280	0.6644	0.5064	−1.7206	3.4852	
**HbA1C vi**	8.0057	26.0020	0.3079	0.7582	−42.9574	58.9688	

### Sensitivity & subgroup analyses

To address the significant heterogeneity, we performed sensitivity and subgroup analyses based on field strength and T1 mapping technique (Supplemental Figs. 1–8). However, neither the sensitivity analyses nor the subgroup analyses were able to resolve the heterogeneity in both the ECV and native T1 analyses. Only the sub-analysis of studies using ShMOLLI showed no significant heterogeneity, possibly due to the small number of studies (three) that used this technique. Therefore, our results should be interpreted with caution.

## Discussion

This meta-analysis sheds lights on the relationship between myocardial fibrosis, as measured by ECV, and diabetes mellitus (DM). Our findings confirm that diabetic patients exhibit significantly higher ECV values compared to controls, indicating an increased degree of myocardial fibrosis. This aligns with previous studies that have identified myocardial fibrosis as a common complication of chronic hyperglycemia, contributing to the increased cardiovascular morbidity in this population [15, 19, 27, 30].

Although ECV values were elevated in patients with diabetes, our analysis did not find a significant difference in native T1 values between these patients and controls, despite a similar trend. This is likely due to the small sample size of the included studies. Therefore, the meta-analysis of native T1 time studies may have lacked sufficient power, as the ECV% meta-analysis included a larger sample size. There are several explanations for the results of the T1 time meta-analysis. First, the native T1 time parameter shows weaker correlation with histology compared to ECV% [13]. Additionally, a cohort study demonstrated that abnormal ECV was more strongly associated with adverse outcomes than native T1 time [33]. Moreover, native T1 time estimates reflect changes across the entire myocardium and may lack sensitivity to detect interstitial-specific alterations [34]. Native T1 time is also more affected by patient characteristics compared to ECV% [35], . For example, in a study where ECV was normal, native T1 times were elevated in patients with diabetes, suggesting a potential influence of diabetes on T1 time parameters independent of MF [25].

Our meta-regression analysis aimed to explore the potential correlation between ECV and HbA1C levels as a measure of diabetes control. However, no significant correlation was found, despite the clear association between higher ECV values and increased variance in HbA1C. The observed lack of significant correlation between HbA1C and ECV may be attributed to several factors. First, the limited sample size of studies reporting HbA1C data for both study arms could result in insufficient statistical power to detect a meaningful relationship. Second, substantial heterogeneity across studies, including variations in patient populations (e.g., diabetes duration, degree of glycemic control) and imaging methodologies, may obscure potential correlations. Variations in study design could also explain the lack of a significant finding. For instance, cross-sectional studies capture data at a single time point, which may not adequately reflect the dynamic relationship between glycemic control and myocardial fibrosis. Furthermore, HbA1C reflects long-term glycemic averages but does not capture acute fluctuations in glucose levels, which might play a more direct role in fibrotic remodeling. Additionally, the mechanisms driving myocardial fibrosis in diabetes are multifactorial and not solely dependent on hyperglycemia. Other factors, such as inflammation, advanced glycation end-products (AGEs), and oxidative stress, could independently contribute to fibrosis, diminishing the observed association between HbA1C and ECV.

Notably, Hajdu et al. (2022) demonstrated a significant correlation between HbA1C levels and strain parameters obtained through echocardiography [36]. This suggests that certain cardiac imaging techniques may be more sensitive to glycemic control’s impact on myocardial function. Differences in imaging modalities, sensitivity to subclinical changes, and study populations could explain these discrepancies. Comparative analyses integrating various imaging methods, such as strain echocardiography and CMR, may help reconcile these findings.

Our findings underscore the complexity of myocardial fibrosis in diabetic patients and the need for more robust and consistent data to better understand the relationship between DM control and myocardial fibrosis. While ECV appears to be a promising marker for assessing fibrosis in diabetic populations, the variability in the association with HbA1C highlights the need for caution in interpreting these results. One potential confounding factor is the reason why CMR was done in different studies. Future studies with larger sample sizes and more consistent reporting of relevant clinical parameters are essential to confirm these findings and refine the use of ECV as a diagnostic tool. If validated, these findings could have clinical significance, as a greater extent of myocardial fibrosis (MF) has been linked to a higher risk of adverse events [21, 32, 37]. Studies utilizing histology to measure MF have shown that each unit increase in collagen volume fraction (CVF) was associated with up to a 28% higher risk of cardiac events and a 50% increase in all-cause mortality. Similarly, for each unit increase in extracellular volume (ECV%), there was a 30% increased risk of recurrent atrial fibrillation (AF) and a 16% higher risk of composite heart failure hospitalization (HHF) or all-cause mortality [21, 32, 37].

In terms of clinical applications, elevated ECV values in diabetic individuals suggest its potential applicability in early detection of myocardial fibrosis, even in asymptomatic stages of the disease. These biomarkers could be incorporated into routine CMR imaging protocols in the future to identify patients at higher risk of adverse cardiac outcomes, such as heart failure or arrhythmias. In clinical practice, ECV mapping may guide treatment decisions by stratifying patients based on the degree of myocardial fibrosis. For instance, patients with significantly elevated ECV values might benefit from more aggressive glycemic control, antifibrotic therapies, or interventions targeting associated metabolic and inflammatory pathways. Additionally, these biomarkers could serve as endpoints in clinical trials evaluating novel therapies for diabetic cardiomyopathy. Risk stratification using ECV could also inform the frequency of follow-up and imaging assessments, prioritizing high-risk patients for more intensive monitoring. Moreover, combining ECV with other clinical data, such as biomarkers of inflammation or endothelial dysfunction, could provide a comprehensive approach to personalized care for diabetic patients.

While native T1 mapping shows potential as a tool for characterizing myocardial tissue properties, the current evidence is inconclusive due to this lack of significance and high heterogeneity across studies. Consequently, its clinical utility requires further validation through standardized protocols and larger studies.

Several limitations should be noted. The studies examining diabetes and myocardial fibrosis (MF) were either cross-sectional or observational, meaning the possibility of reverse causation or bidirectional relationships cannot be ruled out. Additionally, some of the included studies were assessed as being of poor quality. The variability in study quality underscores the need for caution when interpreting pooled results. However, even after excluding these lower-quality studies from our analysis, our findings remained consistent.

For native T1 analysis, the meta-analysis may have been underpowered due to the limited number of studies and substantial heterogeneity. Contributing factors include differences in imaging protocols (e.g., MOLLI vs. ShMOLLI techniques), field strength (1.5T vs. 3T), and patient populations (e.g., age, presence of cardiac comorbidities, glycemic control). Variability in study designs and sample sizes may also contribute. Sensitivity and subgroup analyses failed to resolve the heterogeneity, highlighting the need for further research to better understand native T1’s role in diabetic cardiomyopathy.

To address these limitations, future studies should aim to standardize imaging protocols, including field strength and T1 mapping techniques, to minimize variability. Consistent reporting of patient characteristics, such as diabetes duration and severity, would enhance comparability across studies. Additionally, larger sample sizes and longitudinal study designs could help clarify the relationship between myocardial fibrosis markers and diabetes severity. Addressing these gaps will be crucial for improving the clinical applicability of both ECV and native T1 mapping in diabetic populations.

In conclusion, while our analysis supports the use of ECV as a marker of myocardial fibrosis in diabetics, the relationship between ECV and DM severity remains unclear. Further research is needed to fully elucidate the clinical implications of these findings and to determine the most effective imaging strategies for detecting and monitoring myocardial fibrosis in diabetic patients.

## Supplementary Information

Additional file 1.

## Data Availability

The data and materials supporting the findings of this meta-analysis are available from the corresponding author upon reasonable request.

## Abbreviations

T2DM: Type 2 Diabetes Mellitus
MF: Myocardial Fibrosis
CMR: Cardiovascular Magnetic Resonance
ECV: Extracellular Volume Fraction
HbA1C: Hemoglobin A1C
T1: Native T1 Mapping
WMD: Weighted Mean Difference
CAD: Coronary Artery Disease
HF: Heart Failure
PAD: Peripheral Arterial Disease
AF: Atrial Fibrillation
SD: Standard Deviation
IQR: Interquartile Range
PRISMA: Preferred Reporting Items for Systematic Reviews and Meta-Analyses
NT-proBNP: N-terminal Prohormone of Brain Natriuretic Peptide
LVEF: Left Ventricular Ejection Fraction
HHF: Heart Failure Hospitalization
CVF: Collagen Volume Fraction
