# Supplementary material for: Extracellular volume fraction and native T1 mapping in diabetic cardiomyopathy: a comprehensive meta-analysis
Source: BMC Cardiovasc Disord. 2025 Feb 1;25:70. doi: 10.1186/s12872-025-04496-z (PMC11786336; doi:10.1186/s12872-025-04496-z)
Supplement: Supplementary file 1 — Additional file 1. [file 12872_2025_4496_MOESM1_ESM.docx]

# **Supplementary Table 1. Risk of Bias Assessment of the Included Studies.**

| Study | Selection  (out of 4) | Comparability  (out of 2) | Outcome  (out of 3) | NOS Score | Quality rating |
| --- | --- | --- | --- | --- | --- |
| Al-badri 2018 (14) | 1 | 2 | 3 | 6 | Poor |
| Cao 2018 (15) | 2 | 2 | 3 | 7 | Fair |
| Chirinos 2019 (16) | 2 | 1 | 3 | 6 | Fair |
| Dennis 2020 (17) | 2 | 2 | 3 | 7 | Good |
| Gao 2019 (18) | 2 | 1 | 3 | 6 | Fair |
| Gulsin 2019 (19) | 3 | 1 | 3 | 7 | Good |
| Jiang 2020 (20) | 3 | 2 | 3 | 8 | Good |
| Khan 2020 (21) | 3 | 1 | 3 | 7 | Good |
| Kim 2020 (22) | 2 | 2 | 3 | 7 | Fair |
| Kropidlowski 2020 (23) | 1 | 2 | 3 | 6 | Poor |
| Kucukseymen 2020 (24) | 3 | 2 | 3 | 8 | Good |
| Lam 2019 (25) | 2 | - | 3 | 5 | Poor |
| Laohabut 2021 (26) | 2 | 1 | 3 | 6 | Fair |
| Levelt 2016 (27) | 1 | 1 | 3 | 5 | Poor |
| Liu 2022 (28) | 3 | 2 | 3 | 8 | Good |
| Shah 2013 (29) | 1 | 1 | 3 | 5 | Poor |
| Storz 2018 (30) | 3 | 2 | 3 | 8 | Good |
| Swoboda 2017 (31) | 2 | 2 | 3 | 7 | Fair |
| Wong 2014 (32) | 3 | 2 | 3 | 8 | Good |

The Newcastle–Ottawa Quality Assessment Scale (NOS) for cohort studies. Study quality is categorized based on scores in three domains: (1) **Good quality**: 3–4 in the selection domain, 1–2 in the comparability domain, and 2–3 in the exposure/outcome domain; (2) **Fair quality**: 2 in the selection domain, 1–2 in the comparability domain, and 2–3 in the exposure/outcome domain; (3) **Poor quality**: 0–1 in the selection domain, 0 in the comparability domain, or 0–1 in the exposure/outcome domain. Poor, fair, and good quality studies correspond to high, moderate, and low risk of bias, respectively.


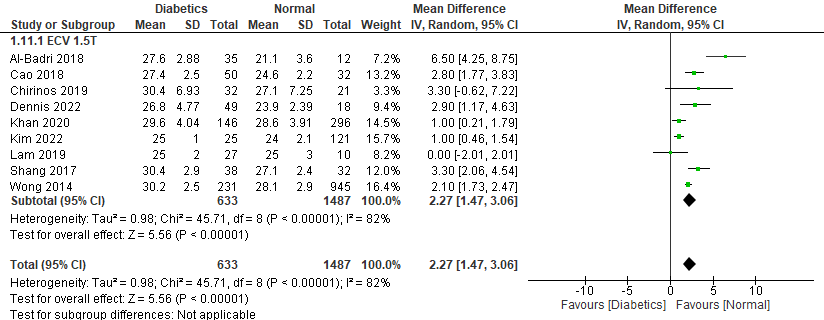


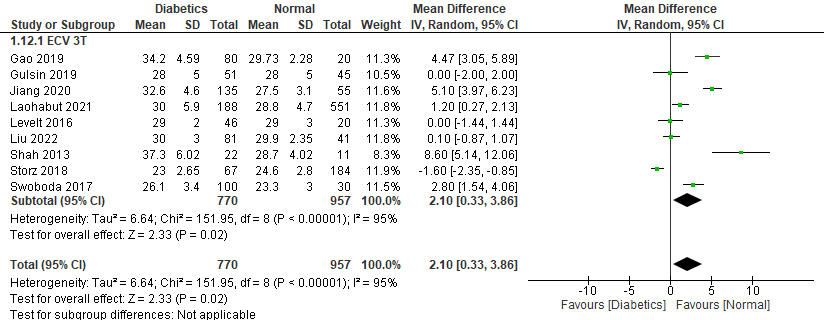


Supplemental figures 1 and 2. Extracellular volume fraction (ECV) subgroup analysis according to field strength.


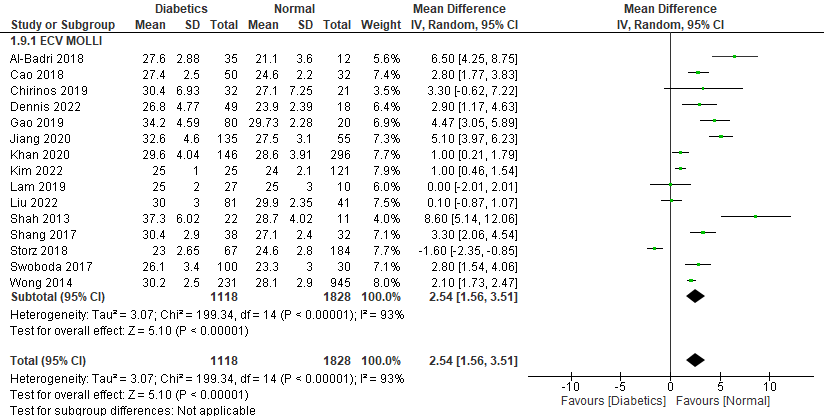


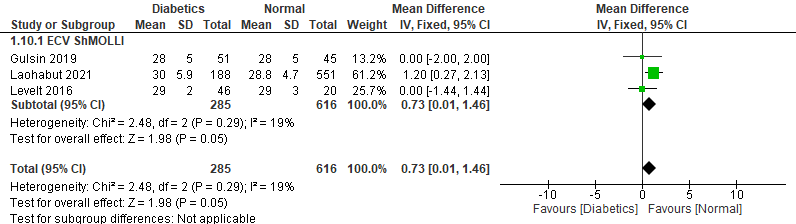


Supplemental figure 3 and 4. ECV subgroup analysis according to T1 mapping technique.


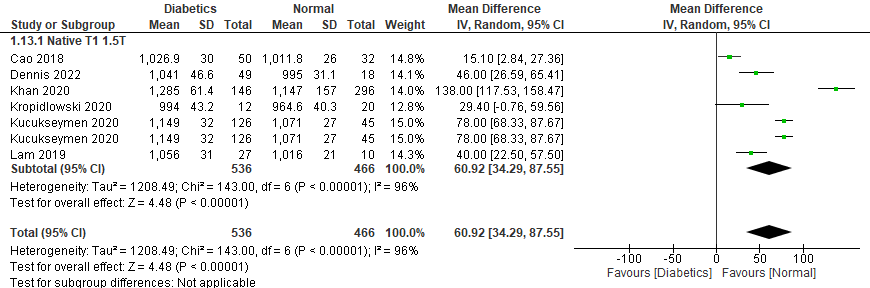


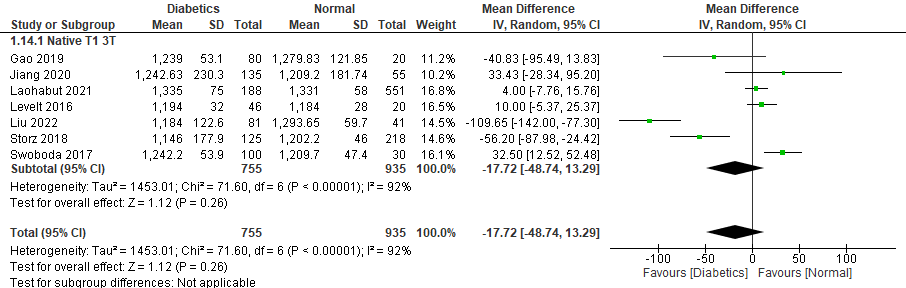


Supplemental figure 5 and 6. T1 subgroup analysis according to field strength.


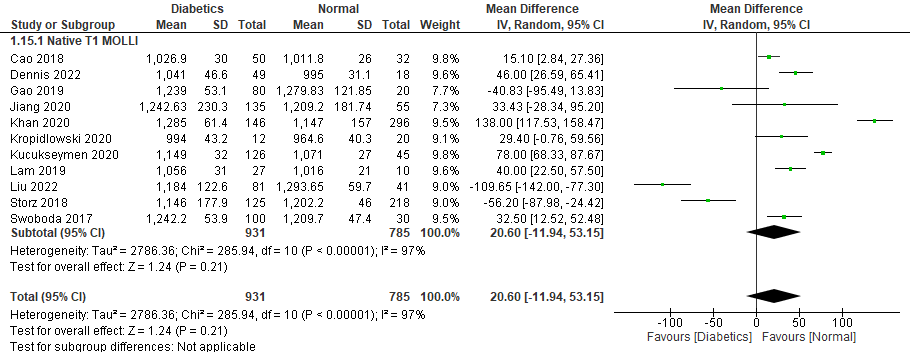


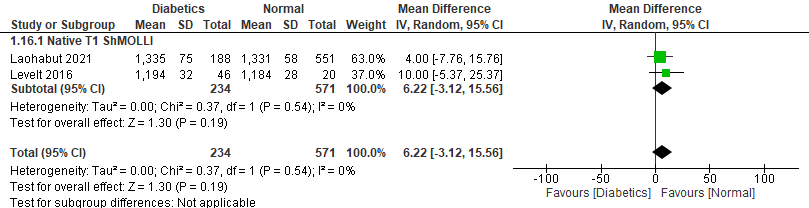


Supplemental figure 7 and 8. T1 subgroup analysis according to T1 mapping technique.
